# Mapping the Mountains of Giants: Anthropometric Data from the Western Balkans Reveal a Nucleus of Extraordinary Physical Stature in Europe

**DOI:** 10.3390/biology11050786

**Published:** 2022-05-21

**Authors:** Pavel Grasgruber, Bojan Mašanović, Stipan Prce, Stevo Popović, Fitim Arifi, Duško Bjelica, Dominik Bokůvka, Jan Cacek, Ivan Davidović, Jovan Gardašević, Eduard Hrazdíra, Sylva Hřebíčková, Pavlína Ingrová, Predrag Potpara, Nikola Stračárová, Gregor Starc, Nataša Mihailović

**Affiliations:** 1 Faculty of Sports Studies, Masaryk University, 625 00 Brno, Czech Republic; bokuvka@fsps.muni.cz (D.B.); jan.cacek@gmail.com (J.C.); hrazdira@fsps.muni.cz (E.H.); hrebickova@fsps.muni.cz (S.H.); nikola@2d.cz (N.S.); 2 Faculty for Sport and Physical Education, University of Montenegro, 81400 Niksić, Montenegro; stevo.popovic@ucg.ac.me (S.P.); dbjelica@ucg.ac.me (D.B.); jovan@ucg.ac.me (J.G.); pid.p9193@gmail.com (P.P.); 3 Western Balkan Sport Innovation Lab, 81000 Podgorica, Montenegro; 4 Gimnazija Metković, 20350 Metković, Croatia; stipan.prce@gimnazija-metkovic.net; 5 Faculty for Sport and Physical Education, University of Tetovo, 1200 Tetovo, North Macedonia; fitim_arifi@yahoo.com; 6 Kosovo Olympic Academy, 10 000 Prishtina, Kosovo; 7 Srednja ekonomsko-ugostiteljska škola (Secondary School of Economics and Catering), 85000 Bar, Montenegro; davidovicivan92@yahoo.com; 8 Department of Anthropology, Faculty of Science, Masaryk University, 602 00 Brno, Czech Republic; ingrova.p@mail.muni.cz; 9 Faculty of Sport, University of Ljubljana, 1000 Ljubljana, Slovenia; gregor.starc@fsp.uni-lj.si; 10 Department of Biostatistics, Institute of Public Health, 34000 Kragujevac, Serbia; natalimihailovic@gmail.com

**Keywords:** Dinaric Alps, Europe, height, genetics, Y haplogroups, GWAS

## Abstract

**Simple Summary:**

The exceptional height of people from the karst area of the Dinaric mountain range has fascinated anthropologists since the end of the 19th century. However, after World War II, little information was available and new representative data started to be collected only at the beginning of the 21st century. Our present work is the most comprehensive summary of the regional differences in body height on the territory of the former Yugoslavia and Albania, and discusses the possible causes of the extraordinary body size of the local population.

**Abstract:**

The inhabitants of the Dinaric Alps (former Yugoslavia and Albania) have long been known as people of impressive body height, but after World War II, there was a critical lack of data related to this phenomenon. This anthropological synthesis includes the measurements of 47,158 individuals (24,642 males and 22,516 females) from the period 2010–2018 and describes detailed regional differences in male stature in the Western Balkans. According to these data, young men from Montenegro (182.9 cm) are currently the tallest 18-year-olds in the world, surpassing their Dutch peers (182.4 cm), and 18-year-old boys from Dalmatia are even taller (183.7 cm) at a regional level. A continuous belt of extraordinary height means (>184 cm) stretches from the Adriatic coast of Dalmatia through Herzegovina to the central part of Montenegro. This article summarizes all the key socio-economic, nutritional, and genetic data, and offers possible explanations for this anthropological phenomenon. Since the remarkable height of the Dinaric populations cannot be connected with any commonly known environmental factor, the most probable hypothesis is genetic and links these physical characteristics with the local founder effect of Y haplogroup I-M170. Furthermore, given that both the level of socio-economic development and dietary protein quality are still sub-optimal, the local upward trend in body height has the potential to continue in the future.

## 1. Introduction

**Figure 1:**
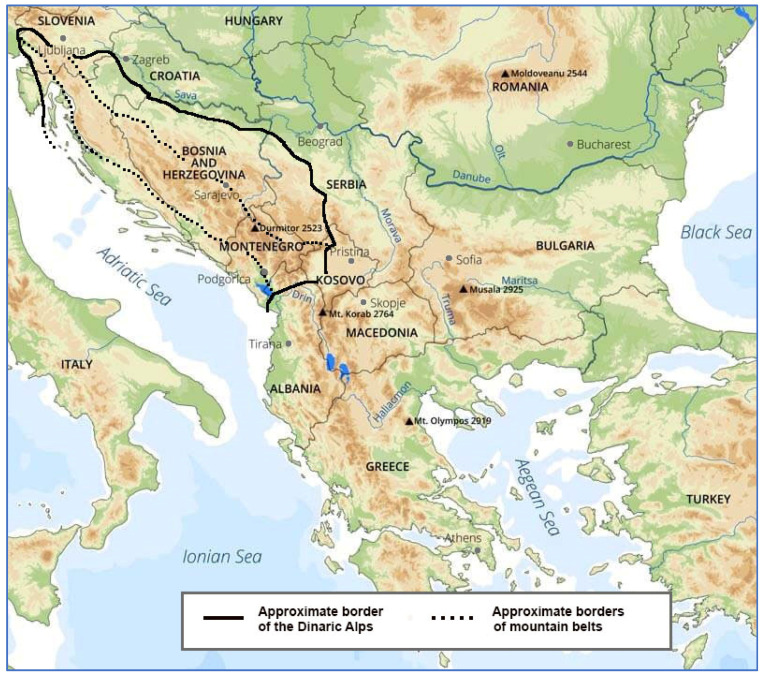
Approximate border of the Dinaric Alps.

The exceptional height of people from the karst area of the Dinaric mountain range (Figure 1) has attracted the attention of leading anthropologists since the end of the 19th century. At that time, the height of males in Bosnia and Herzegovina ranged from 171 cm in the region of Tuzla to 175–176 cm in Herzegovina [1], and local young men serving in the Austro-Hungarian army in 1895 were 172.4 cm tall [2]. Similar-aged cohorts from Northern Europe reached only ~170 cm, and Western and Central Europeans were even shorter [3]. In fact, the average height of Austro-Hungarian recruits measured in 1890 was only 165.1 cm. Not too surprisingly, Croatians were by far the tallest at 167.8 cm [4]. After World War I, the Czech researcher Valšík [5] performed an anthropological survey in the Durmitor mountains of northwestern Montenegro and documented a mean height of 176.7 cm in 163 males aged 18–40 years—a value that the highly industrialized nations of Northern and Western Europe started to approach only during the 1950s and 1960s [3]. It is no wonder that prominent Harvard anthropologist Carleton Coon devoted an entire book (*The Mountains of Giants, 1950*) [6] to the research of mountaineers from northern Albania. Coon found large differences in height between Dukagin men from the northernmost tip of the Shkodër county (173–174 cm) and men from the Mat river basin in the Lezhë county (167 cm), and speculated that body size is positively associated with minerals from the limestone bedrock. In contrast, short-statured Albanians lived mainly on flysch (sandstone). 

After several decades, when no detailed anthropometric data from the Dinaric Alps were available, the study of Pineau et al. [7], undertaken between 2001–2003 in Dalmatia and Herzegovina, again drew deserved attention to this phenomenon. Nevertheless, this study did not cover all regions and the impressive male mean of 185.6 cm was speculatively elevated by adding +1 cm to the presumably unfinished growth of 17-year-old boys. Although at least two other anthropometric surveys were subsequently performed in Bosnia and Herzegovina [2,8], they were small and covered only cantons of the Federation (inhabited by Muslims/Bosniaks and Croats). To overcome this long gap and explore the current state of the secular height trend, a common JoinEU-SEE Project of Masaryk University in Brno (Czech Republic) and the University of Montenegro decided to focus on the detailed anthropological mapping of the whole Dinaric area: Bosnia and Herzegovina, coastal Croatia, Montenegro, and the territory of Kosovo. This complex approach was substantial, due to the high regional variability and rapid geographical changes of height means. Our planned research in Albania was eventually cancelled because detailed regional data are already available through the Albania DHS (Demographic and Health Survey) 2017–2018 [9]. 

The aim of the present article is to make a comprehensive synthesis of the work of both universities and to put it into a broader context, using other anthropological studies from the Western Balkans. The combination of these data should create a detailed picture of the regional variation in body height in this region. The article will also summarize scientific evidence related to this interesting anthropological phenomenon, and discuss its possible interdisciplinary implications.

## 2. Methods

The collaboration between the Faculty of Sports Studies (at Masaryk University in Brno) and the Faculty for Sport and Physical Education in Nikšić (affiliated with the University of Montenegro) started in 2012, when the latter institution prepared a nationwide survey of Montenegrin high school students aged 17–20 years. This survey was conducted in 2013 [10] and it was agreed that the research would be extended to other areas of the Dinaric Alps, utilizing the JoinEU-SEE mobility and scholarship project between the EU and the Western Balkans. In 2016, the University of Montenegro finished its research in Kosovo [11], whereas Masaryk University covered Bosnia and Herzegovina (2015–2016) [12] and coastal Croatia (2015–2017) [13], using the help of a Croatian high school teacher (S. Prce). Due to time constraints, the research of Masaryk University concentrated on young men, and female samples are thus mostly small. 

All these surveys were first approved by the ethics committees of the involved universities, and by the local ministries of education. The final approval depended on individual school directors and their agreement with the students’ parents. Detailed measurement procedures and analyses of results are described in the individual research reports and were performed in accordance with relevant guidelines. To ensure maximal representativeness, the surveys included a broad spectrum of school types, from elite high schools *(gimnazije)* to vocational schools *(strukovne škole)*, and targeted similar age groups with finished or nearly finished growth (17–20 years). Although the sample from Kosovo did not include 17-year-olds, the mean age of measured young men was practically identical (18.3 ± 0.3 years in Bosnia and Herzegovina, 18.5 ± 0.6 years in coastal Croatia, 18.4 ± 0.6 years in Montenegro, and 18.3 ± 0.5 years in Kosovo). 

To obtain a more complex anthropological picture of the Western Balkans, we also included regional anthropometric data from the Albania Demographic and Health Survey (Albania DHS) 2017–2018 [9], the Serbia National Health Survey (Serbia NHS) 2013 [14], and the annual measurements of youth in Slovenia (a mean for 2015–2017) (G. Starc, personal communication). Because the number of measured individuals in the Albanian and Serbian survey was too small, the age range in both surveys was extended to 17–25 years. No regional data were available for North Macedonia [15], although its size is comparable to that of the major statistical regions in Serbia. Out of all regions of the former Yugoslavia, only mainland Croatia was represented poorly—by the Karlovac county (which was included in ‘Coastal Croatia’) and by a small survey from Zagreb [16]. 

## 3. Results

**Figure 2:**
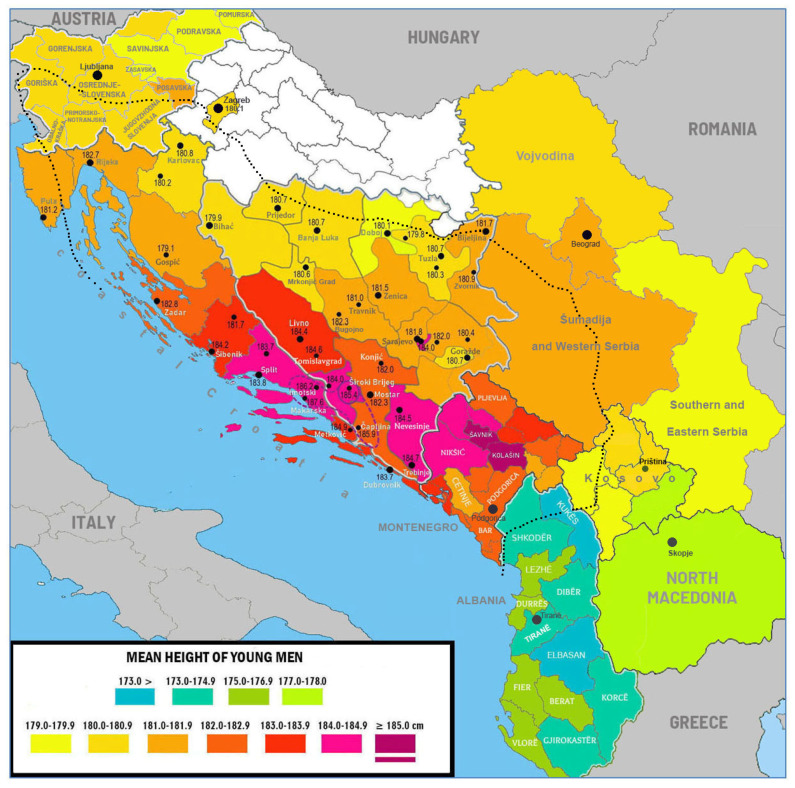
Regional differences in male height in the former Yugoslavia and Albania, including the means of individual towns in Croatia and Bosnia and Herzegovina. The data are based on studies listed in Table 1. For Zagreb, see Petranović et al. [16]. The interrupted violet line demarcates an area with urban means above 185 cm. The approximate border of the Dinaric Alps is shown by a dotted line.

The common project of both universities, which consisted of four surveys (from Bosnia and Herzegovina, coastal Croatia, Kosovo, and Montenegro), included anthropometric measurements of 9557 individuals (6806 boys and 2751 girls) aged ~18 years on average (with the maximum age range of 17–20 years) (Table 1). Together with the surveys from Albania, mainland Croatia (Zagreb), North Macedonia, Serbia, and Slovenia, the total number of measured subjects in our study reached 47,158 (24,642 males and 22,516 females). The graphical representation of the results (Figure 2) demonstrates both exceptionally high values of stature and striking interregional differences that have few parallels in other parts of the world. The area with the tallest male statures (above 184 cm) is a continuous belt stretching across three countries and very different landscapes—from the Adriatic coast of Dalmatia through Herzegovina to the central mountainous municipalities of Montenegro. This belt also includes two notable regional anomalies with mean heights above 185 cm. One is centered around Široki Brijeg in Western Herzegovina and Central Dalmatia, where a small urban sample of boys from Makarska reached 187.6 cm (*n* = 27). The other is in Central Montenegro, in the sparsely populated municipalities of Kolašin and Šavnik (185.5 cm, *n* = 30). Despite the small sample size, this result is hardly accidental, as this region includes the Durmitor mountains in northern Šavnik, where Valšík [5] once reported his record mean of 176.7 cm. Exceptional Dinaric height in a wider sense (>181 cm) extends to Istria in the west and to Šumadija and Western Serbia in the east. 

The average male height in Dalmatia (183.7 cm), Herzegovina (183.4 cm), and Montenegro (182.9 cm) is close to that of Dutch men aged 21 years (183.8 cm), who are regarded as the tallest in the world [17] (Table 2). Information on Dinaric girls is more limited but their average height in these regions is approximately 169 cm—lower than that of young Dutch women aged 21 years (170.7 cm). However, it is worth noting that education in the Netherlands is compulsory up to the age of 16–18 years and data on older subjects were obtained outside the general school system, which increased the risk of selection bias. In fact, Dutch boys and girls aged 18 years are only 182.4 cm and 169.7 cm tall, respectively. Therefore, although Montenegrin women are definitely shorter than Dutch women, it is possible that young Montenegrin men are actually taller than Dutch men. Similarly, Dalmatian boys probably surpass their peers in the northern Netherlands at the regional level [13]. 

The gradient of decreasing height from the ‘core area’ of the Dinaric Alps was mostly very steep, especially across the main mountain range of the Dinaric Alps in Bosnia and Herzegovina. For example, large town samples in the cantons of Livno and Western Herzegovina consistently reached 184–185 cm, whereas boys residing in the town of Mrkonjić Grad, situated on the opposite side of the mountain barrier, were only 180.6 cm tall. These rapid changes cause large inter-regional differences within individual countries. The sharpest polarity of this sort could be observed in Bosnia and Herzegovina, between the region of Doboj (179.7 cm) and the region of Trebinje (184.5 cm) (a difference of 4.8 cm). In Montenegro, boys from the municipalities of Kolašin and Šavnik reached 185.5 cm, whereas boys from the municipality of Cetinje (a former Montenegrin capital) were only 181.3 cm tall (4.2 cm). The height of boys on the Adriatic coast of Croatia also varied widely—from 180.6 cm in the county of Karlovac to 184.1 cm in the county of Split (3.5 cm). 

The regional means from Serbia, Slovenia, and the territory of Kosovo (which lie largely outside the main Dinaric mountain range) were more homogeneous in this respect, with differences of only ~2 cm across regions. Still, the decrease in height—in the direction from the central part of the Dinaric Alps—was clearly visible, especially towards North Macedonia. The rapid drop in the southwestern part of the mountain range concerned even Albania, which constituted a very special case—first, its regional data did not fit well into the geographical trends in the Western Balkans, and second, the difference in the mean height between Montenegro and Albania reached unusually large dimensions. According to the Albania DHS survey 2017–2018 [9], young Albanian men aged 17–25 years were only, on average, 174.4 cm tall, which means that they are among the shortest in Europe, 8.5 cm shorter than their Montenegrin peers. To our knowledge, only the differences across the border of North and South Korea (8.8 cm), and the USA and Mexico (8.6 cm) are comparable (see [18]). Similarly, Albanian women were 7.2 cm shorter. Furthermore, the drop in male height between the regions of Kukës (Albania) and Kolašin and Šavnik (Montenegro) was ~13 cm over a distance of 50 km.

## 4. Discussion

### 4.1. Factors Associated with Dinaric Tallness

The findings of this research naturally raise many questions. The most fundamental one is whether this local phenomenon can be explained by some environmental factors, or if we should look for exceptional genetic predispositions of the local population. Height is, by nature, a highly heritable trait [19], but it is also a very sensitive indicator of living conditions and the role of environment can be huge. Indeed, following the Industrial Revolution, the height of European nations has increased by 10–17 cm since the end of the 19th century [3,20]. Detailed analyses of this process have been performed e.g., for Germany [21], Norway [22], and the United States [23]. This increase in stature is driven by several key factors that are closely associated with the rising GDP (gross domestic product) per capita: better nutrition (mainly high-quality proteins from milk, pork, and eggs), declining rates of total fertility (which determine the distribution of resources within families), the absence of infectious diseases that exhaust the growth capacity of the child’s body (which is reflected in lower child mortality), urbanization (which facilitates better access to resources and healthcare), and social equality [3,20]. 

Previous studies [12,13,18,20] have shown that when viewed from the perspective of these major environmental factors, the height of the Western Balkan countries is a striking anomaly, in both the European and global context. In fact, a regression model of six socio-economic and three nutritional variables in 119 countries [18] showed that the predicted male height for Bosnia and Herzegovina (173.5 cm) deviated the most by far (by 7.7 cm) from the true, observed height (181.2 cm), and it was even lower than the predicted height for Albania (177.0 cm). The positive residual in the case of Serbia (5.1 cm) was the third highest in this global sample (176.1 cm vs. 181.2 cm) (after Haiti, which is heavily dependent on international aid). These numbers remain practically the same even in a smaller sample of 96 countries, after the addition of the Gini index (a measure of social inequality) and the replacement of HDI (Human Development Index) with IHDI (inequality-adjusted HDI): 7.4 cm for Bosnia and Herzegovina and 5.1 cm for Serbia. 

**Figure 3:**
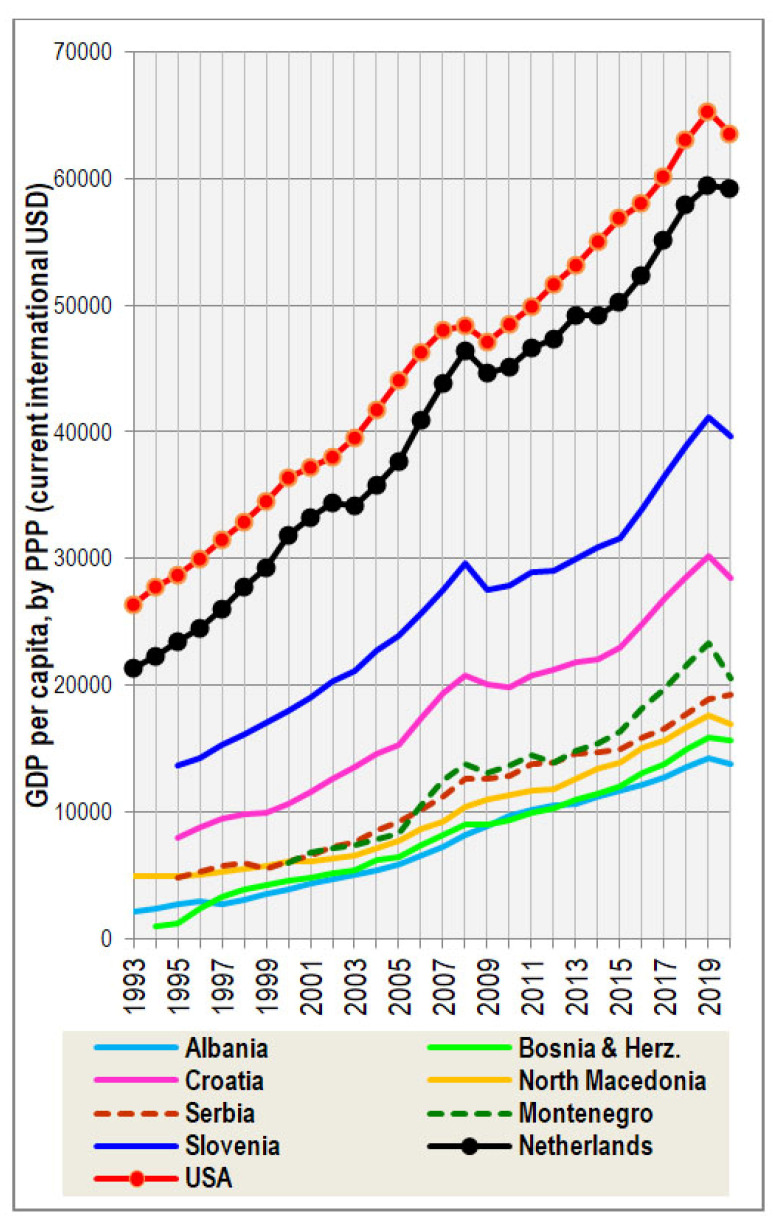
Economic development in the Western Balkans (gross domestic product per capita, by purchasing power parity), compared with the Netherlands and the USA. Source: [24].

**Figure 4:**
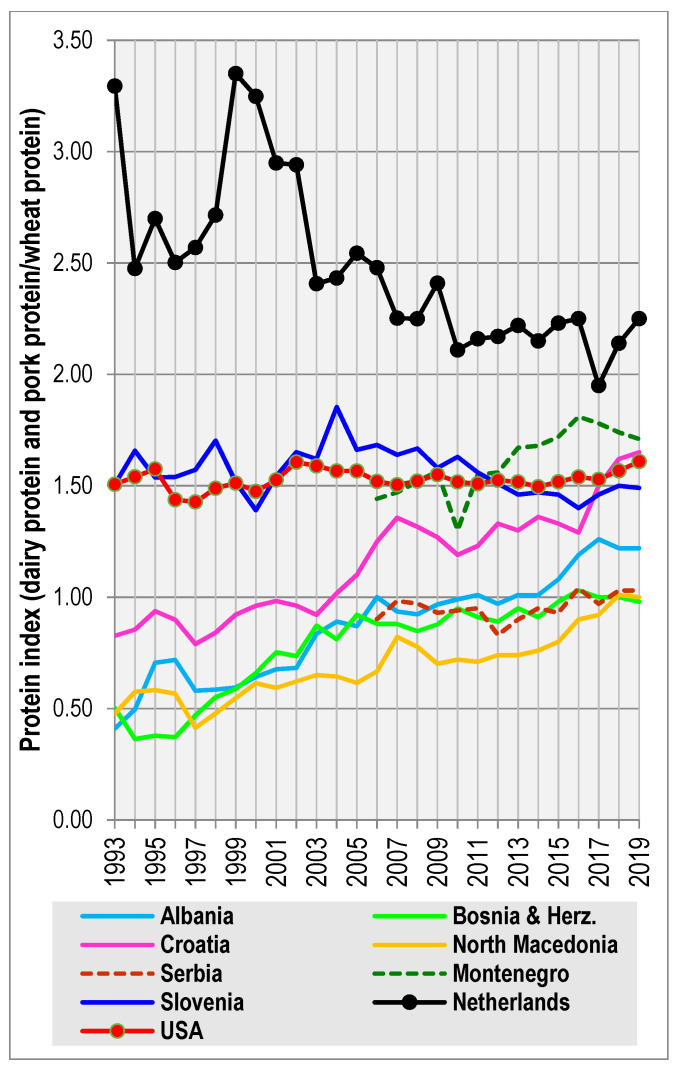
The quality of nutrition in the Western Balkans (expressed as the ‘protein index’), compared with the Netherlands and the USA. Source: [25]. Note: Data for 2010–2019 were computed using the new FAOSTAT methodology.

These eccentric results are easy to understand, when we consider that the GDP in the Netherlands for 2013 was 49,242 USD per capita, whereas that of Montenegro was more than three-times lower (14,870 USD per capita), and the GDP in Bosnia and Herzegovina reached only 12,011 USD per capita in 2015 [24] (Figure 3). Even more important are the statistics of dietary protein quality, assessed using the FAOSTAT database [25]. Indeed, the ‘protein index’ (a ratio between the daily supply of proteins from dairy and pork/wheat), which is the strongest nutritional predictor of height in 44 European countries (*r* = 0.62, *p* < 0.001; see [18]), is mostly very low in the Western Balkans (Figure 4). In any case, it is far below the level of the Netherlands, which has been, until recently, characterized by the highest dietary protein quality in the world. Only Montenegro and Slovenia were among the top 15 ranked European countries between 2010 and 2019, and Croatia got above the European mean only very recently. What is even more stunning is the fact that the generation surveyed in the Dinaric Alps was often growing up in very difficult conditions during a post-war period, when these protein indices were substantially lower.

The knowledge of these dietary factors can illuminate the unexpectedly short stature of local Muslims in Bosnia and Herzegovina, who do not consume pork for religious reasons [12]. This paradox is particularly apparent in the capital of Sarajevo, where we observed a 2.2 cm difference (181.8 cm vs. 184.0 cm) between the major Muslim part of the city and the eastern Serbian part (Istočno Sarajevo). This regional anomaly also occurred in the Muslim enclave of Goražde, and in the Muslim part of Herzegovina between Konjic and Mostar, where we found 2–3 cm shorter averages than in the neighboring Croatian and Serbian regions.

**Figure 5:**
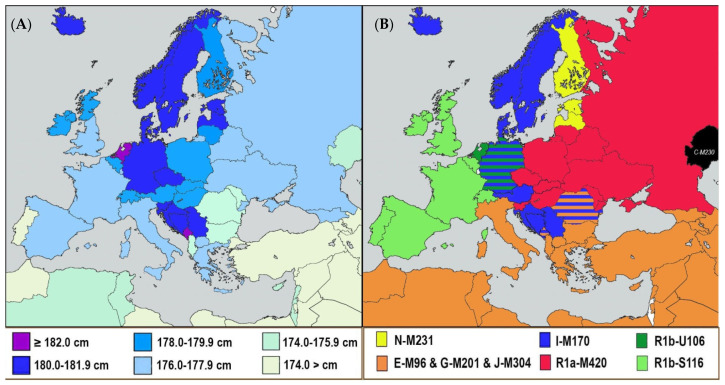
(**A**) The actual distribution of male height in Europe. (**B**) The most frequent Y haplogroups (or their combination) in European nations. The frequencies of Y haplogroups that do not differ by more than 1% are indicated by hatching.

However, the only variable that meaningfully explains Dinaric height as a whole is genetic—the frequencies of Y haplogroup (male lineage) I-M170. Our updated data (Grasgruber et al.—in review) show that this haplogroup correlates strongly positively with male height both within the seven countries of the Dinaric Alps (*r =* 0.80, *p =* 0.030) and within 55 countries of Europe and the Near East (*r =* 0.73, *p <* 0.001). In contrast, Y haplogroups of Near Eastern origin (E-M96, G-M201, J-M304) predict short stature in the Dinaric Alps (*r* = −0.70, *p* = 0.079), and this is especially true for J-M304 (*r* = −0.88, *p* = 0.008), which is also the strongest correlate of shortness in Europe and the Near East (*r* = −0.86, *p* < 0.001). In accordance with these findings, the inclusion of I-M170 and the three Near Eastern Y haplogroups dramatically improves the best regression model of male height in Europe: from adjusted R^2^ = 0.426 to 0.721 [18]. Other European Y haplogroups have a more restricted geographical distribution but they also show geographical relationships with height (see Figure 5A,B and [20]). 

Y haplogroup I-M170 generally constitutes a very interesting case because its origin is very old and can be traced as far back as to the Upper Paleolithic Gravettian culture [26]. I-M170 was also closely associated with the post-glacial expansion of Epigravettian (Late Gravettian) groups from the refugium around the Adriatic Sea and became the predominant Y haplogroup in Mesolithic Europe [27]. At present, it reaches a global frequency peak in Herzegovina (70.9%) [28], where it is overwhelmingly represented by the Balkan sub-branch I2a1a-P37.2 and particularly by downstream mutations of I2a1a2-M423 [29]. This dominance of I2a1a2-M423 would result from a relatively recent (possibly even post-Neolithic) founder effect [28,29,30,31]. Still, the history of I-M170 in the Dinaric Alps remains enigmatic and due to the scarcity of well-preserved skeletal material, it will be difficult to capture its evolution over time. In fact, the website of Ancient Human DNA [32] does not register a single prehistoric sample from Bosnia and Herzegovina and Montenegro [32]. To our knowledge, the oldest occurrence of I-M170 (I2a1a2-M423) in the Dinaric area was documented in the Bezdanjača Cave (Lika-Senj County, Adriatic Croatia) and was indirectly dated to ~1200 cal. BC [33]. A more recent analysis of Bronze Age individuals from this cave reportedly found another case of I2a1a2-M423 (I. Lazaridis, personal communication). However, Utevska [34] doubted the local origin of I2a1a-P37.2 in theWestern Balkans and hypothesized that it had expanded ~3000 years ago from the area east of the Carpathians.

**Figure 6:**
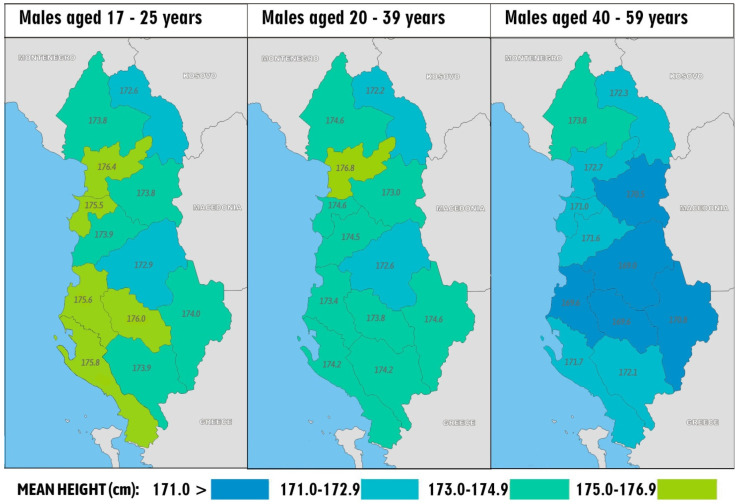
Regional differences in male height within Albania, according to different age categories: 17–25 years (*n* = 1273), 20–39 years (*n* = 2502), and 40–59 years (*n* = 2072). Source: [9].

In any case, the genetic explanation would fit the distribution of height in the Dinaric Alps because the area with the tallest statures does not follow the border of the limestone bedrock (as hypothesized by Coon), but rather, it lies deeper behind the main mountain range that served as a barrier to the genetic flow from the European mainland. Environmental explanations fail especially in Montenegro, where people in the richer coastal regions around the capital of Podgorica are much shorter than those in the remote mountain areas of Kolašin and Šavnik lying mostly on flysch sediments [35]. Only Albania is an exception because here, we observe a completely different geographical trend between the short-statured mountainous hinterland (especially Elbasan and Kukës) and the taller Adriatic coast. Age-stratified regional data from the Albania DHS 2017–2018 (Figure 6) indicate that the main cause of this anomaly must be sought in Albania’s long-term economic stagnation and isolation. In other words, height in many Albanian regions has long been conserved at the medieval level (~170 cm in men) due to deep economic underdevelopment, and it is natural that the improvement of living conditions during the last three decades was accompanied by a very fast positive trend in the most affluent coastal counties. In contrast, Elbasan and Kukës remained the poorest counties in Albania [36].

### 4.2. Current Genomic Evidence

Given that Y haplogroups are uniparental markers that best serve the purpose of genetic genealogy and do not accurately reflect autosomal ancestry, the genetic explanation of the Dinaric phenomenon requires an analysis of autosomal DNA that would identify specific genetic loci linked to the selection for height. Comparisons of this sort are already available thanks to genome-wide association studies (GWAS) and have shown that height is highly polygenic—depending on the combination of a large number of genes, each of which explains only a very small part of the total variability [37,38]. At present, hundreds of potentially height-associated SNPs (single nucleotide polymorphisms) are known, and most of them were reported by the GIANT consortium (Genetic Investigation of Anthropometric Traits). Their combined effect (polygenic height scores) is also used as the predictor of height at the population level. However, the identification of such SNPs is difficult due to the confounding role of environment [39]. Although the accuracy of the polygenic height scores has been improving at the individual level [40], their success at the population level is still rather mixed because height-associated SNPs are population-specific and most GWAS have been performed on Europeans [41,42]. Another drawback of these studies is that they pay virtually no attention to the Western Balkans. As a result, the number of publicly available individual genomes from the Western Balkans is relatively small and considering that they are not sorted according to regions in the POPRES database [43], a direct testing of the genetic hypothesis was not possible.

Contemporary genetic studies aimed at the evolution of height in Europe (e.g., [39,41,44]) touched the Western Balkan region at best only superficially and are not very helpful either. Their results generally predict tall statures in ancient populations emerging out of the Epigravettian refugium (genetic cluster Villabruna/WHG) and in the nomadic Eneolithic cultures from the East European steppe (genetic cluster Yamnaya), and attribute low genetic predispositions to Near Eastern agriculturalists. This result agrees with the Y chromosomal picture but curiously, polygenic height scores in the modern Western Balkan populations are only moderate or below average. For example, the preprint by Berg et al. [41] estimated medium height in present-day Croats and predicted the highest values in Icelanders, Englishmen, and Scots. At the same time, English and Scottish males reach only ~178 cm, despite a long historical lead in industrial development, whereas Croats are almost 3 cm taller. This discrepancy raises legitimate questions about the accuracy and population-specificity of these predictions. A later study by Sohail et al. [39] used presumably unconfounded SNPs based on British individuals from the UK Biobank, but these markers also produced only low-to-medium polygenic scores in a small POPRES sample from the former Yugoslavia (*n* = 44). This finding is even more problematic because height means in the Western Balkans are among the highest that have ever been documented in any human group, and five countries of the former Yugoslavia are among the top 13 tallest in the world. Therefore, it is very difficult to imagine that the local populations would be endowed with below-average genetic predispositions in the European context. An alternative explanation must work with the possibility that polygenic height scores derived from Western Europeans are not usable for the Western Balkans and the genetic variation in height in Europe is underestimated.

### 4.3. Future Perspectives

The suboptimal socio-economic and nutritional statistics summarized in the present study suggest that the region of the Western Balkans has not yet reached its maximum potential in terms of physical stature. In fact, the projection of correlation lines between male height and the frequencies of I-M170 indicates that well-nourished males in Herzegovina and southern Dalmatia could potentially reach an astonishing average height of ~190 cm [12,13]. This value, however seemingly improbable, is already not too far from the urban means that we documented in Makarska (187.6 cm), Imotski (186.2 cm), and Čapljina (185.9 cm). The current development of dietary protein quality appears the most optimistic in the case of Croatia and Montenegro (Figure 4). Judging from the measurements of recruits in the capital of Podgorica born during the 1960s [45], the height of Montenegrin men has been increasing at a rate of 1.7 cm/decade. Croatian boys measured during nationwide surveys in 1980–1984 and 2006–2008 grew by 2.9 cm [46], until their growth stopped (or even reversed) during the economic recession in the late 1990s [47]. The current positive trend in the dietary protein quality predicts that their height should increase again. 

The most interesting situation can be observed in Albania, where the ‘protein index’ has been rising relatively fast and steadily since the early 1990s. Provided that all the lagging Albanian regions reach sufficiently high nutritional and socio-economic standards, we expect that the north-to-south gradient in height, which was once reported by Coon [6], will emerge again, and the height difference between Albania and Montenegro should also decrease. The future potential of men from northern Albania can probably be best illustrated by the example of contemporary men from Western Kosovo. However, as already mentioned, the drawback of the Muslim diet in Albania and Bosnia and Herzegovina lies in the negligible consumption of pork, whose nutritional value is not easy to replace. Given that Albanians already consume the highest amount of dairy proteins in the world [25], the values of the ‘protein index’ in Albania are unlikely to rise much higher. 

In Slovenia, we observed only negligible increases in height means in annual school surveys (G. Starc, personal communication), which is in accordance with the recent stagnation of the dietary protein quality. Bosnia and Herzegovina and Serbia have recovered from the negative consequences of the Balkan wars (1991–1999), but the consumption of high-quality proteins in these two countries is still rather limited. Nevertheless, 18-year-old boys from the high schools in Tuzla (Bosnia and Herzegovina) grew by 2 cm between 1980 and 2003, despite war hardships [48], and judging from our data, their height has further increased from 178.8 cm in 2003 to 180.5 cm in 2015. This is a remarkable pace of the secular trend, given the fact that the diet of Bosnian Muslims does not include pork, which is much more consumed in Herzegovina. The quality of nutrition in North Macedonia is similarly low and it is noteworthy that the height of young men was 4 cm lower than in neighboring Serbia. The high share of Albanians in the total population (~25%) could be one of the reasons, but the relatively short mean height documented in Southern and Eastern Serbia, as well as in southern Kosovo, testifies that this tendency towards shorter statures is typical of the whole area and stems from genetic factors. 

### 4.4. Dinaric Populations Outside the Balkans

During the Balkan wars, hundreds of thousands of Muslims (Bosniaks) from Bosnia and Herzegovina have taken refuge in wealthy western countries—especially in the United States, German-speaking countries, and Scandinavia. Given the insufficient and conflicting evidence from genetic studies, it could be very illuminating to compare the height of the immigrant youth with the height of the young population in their host countries. Assuming that both groups were exposed to a similar lifestyle and environment, their mutual comparison could bring important information about their genetic potential. Unfortunately, so far we have not been able to obtain any representative data of this kind. Although a decent sample (107 boys and 109 girls from the “former Eastern Europe”) was reportedly measured during the ‘Grow-Up Gothenburg’ study in Sweden (J. Chaplin, personal communication), the authors of this research did not want to collaborate. We hope, therefore, that our article will serve as an incentive for other researchers in these countries.

## 5. Conclusions

Our research in the Dinaric Alps has filled a long, century-old gap in the information on the secular trend in body height in the region. Including the newly analyzed data from Albania, Serbia, and Slovenia, we obtained an overview of the regional variability in stature over an area of ~230,000 km^2^, across eight different countries or territories. The results demonstrated that, together with the Dutch, some of the local populations are the tallest in the world, and anthropometric data matched for age even suggest that Montenegro is the country with the absolute tallest population. Our article tried to summarize all possible explanations of this interesting anthropological phenomenon, out of which the genetic one, associated with the founder effect of Y haplogroup I-M170, definitely appears as the most likely. The height of the Dinaric populations is not related to any commonly known socio-economic factor, and with the exception of Montenegro and Slovenia, the quality of nutrition has also been suboptimal in the recent past. Furthermore, the generation measured in our studies was often growing up in difficult post-war conditions. Given that some of the regional averages of height are among the highest that have ever been recorded in a human population, it is very difficult to postulate the existence of a strong environmental variable that is still unknown to science and that would be able to counterbalance all these negative factors. What is even more remarkable is the fact that with the improvement of living conditions, a continuation of the secular height trend in the Western Balkans can be expected in the future. 

Still, the contradiction between this genetic explanation and the persisting lack of any genetic evidence at the autosomal level is striking and requires further research. Provided that polygenic height scores based on Western Europeans fail as a predictor of height in the Dinaric Alps, they would not be the best tool for the evaluation of the genetic variability in Europe. This could potentially have important practical implications for the research of polygenic genetic traits. A definitive answer to this problem would require a sophisticated genetic study, which would include both prehistoric and modern samples from the Western Balkans. 

## Figures and Tables

**Table 1:** Mean height in the former Yugoslavia and Albania (in similar age categories).

Country/Region	Year	Age	Males				Females			
			*n*	Mean Height (cm)	SD	SE	*n*	Mean Height (cm)	SD	SE
**Montenegro**	**2013**	**17–20**	**981**	183.4 (182.9 *)	**6.9**	**0.22**	**1107**	169.4 (168.8 *)	**6.4**	**0.19**
*Central region*			664	183.6	7.0	0.27	711	169.7	6.3	0.24
**Coastal Croatia**	**2015–2017**	**17–20**	**1803**	182.7 (182.6 *)	**6.7**	**0.16**	**782**	167.4 (168.0 *)	**6.2**	**0.22**
*Dalmatia*			1143	183.6 (183.7 *)	6.7	0.20	*279*	168.8 (168.5 *)	6.0	0.36
**Bosnia & Herzegovina **	**2015–2016**	**17–20**	**3192**	181.7 (181.2 *)	**6.8**	**0.12**	**69**	**169.4**	**6.0**	**0.72**
*Bosnia*			2209	180.9 (180.8 *)	6.5	0.14				
*Herzegovina*			983	183.6 (183.4 *)	6.9	0.22				
**Serbia**	**2013**	**17**–**25**	**724**	180.7 (180.7 *)	**7.4**	**0.27**	**787**	166.8 (166.8 *)	**6.4**	**0.23**
*Beograd*			155	181.8	7.3	0.59	184	168.3	6.5	0.48
*Šumadija* & *W. Serbia*			225	181.1	7.6	0.51	225	167.4	6.2	0.41
*Vojvodina*			154	180.2	7.4	0.60	194	166.1	6.2	0.45
*South* & *East Serbia*			190	179.8	7.3	0.53	184	165.2	6.5	0.48
**Slovenia**	**2015–2017**	**18**	**15,112**	**180.2**	**6.8**	**0.06**	**15,429**	**166.9**	**6.1**	**0.05**
**Croatia: Zagreb**	**2010**	**18–19**	**131**	180.1 **			**111**	165.2 **		
**Kosovo**	**2016**	**18–20**	**830**	**179.5**	**6.0**	**0.21**	**793**	**165.7**	**4.9**	**0.17**
**North Macedonia**	**2012**	**18**	**596**	**177.4**	**6.5**	**0.27**	**552**	**164.5**	**6.2**	**0.26**
**Albania**	**2017–2018**	**17–25**	**1273**	174.3 (174.4 *)	**6.7**	**0.19**	**2886**	161.5 (161.6 *)	**6.5**	**0.12**

SD: standard deviation; SE: standard error. * A weighted mean considering the population size of individual regions. ** A weighted mean of 18 and 19-year-olds in Zagreb.

**Table 2:** Mean height by country (top 15 tallest countries). Countries of the former Yugoslavia are highlighted in bold. Note: The mean height of young women in Bosnia and Herzegovina was not measured but it should be similar to that of Serbian women (~167 cm). This list does not include sub-Saharan Africa but with the possible exception of South Sudan, there are no countries that could influence this ranking. For sources, see [18].

Males					Females				
Country/Region	*n*	Age	Date	Height	Country/Region	*n*	Age	Date	Height
Netherlands	*	21	2009	183.8	Netherlands	*	21	2009	170.7
**Montenegro**	**981**	**17–20**	**2013**	**182.9**	**Montenegro**	**1107**	**17–20**	**2013**	**168.8**
Iceland (Reykjavik)	146	18	2008–2009	181.8	Lithuania	255	18	2012	168.4
Estonia	644	18–19	2006–2009	181.5	Estonia	927	18–19	2006–2009	168.2
Sweden (Göteborg)	2408	17–20	2008–2009	181.4	Denmark	315	18–24	2007–2008	168.1
**Serbia**	**1072**	**20–29**	**2013**	**181.2**	Iceland (Reykjavik)	129	18	2008–2009	167.9
**Bosnia and Herzegovina**	**3192**	**17–20**	**2015–2016**	**181.2**	Sweden (Göteborg)	2188	17–20	2008–2009	167.9
Denmark (conscripts)	31,056	~18–26	2015	180.7	Latvia	636	20–29	2014	167.3
Czechia (Brno)	1239	18–19	2015–2016	180.6	Hungary	6093	18–25	2016	166.9
**Croatia**	**358**	**18**	**2006–2008**	**180.5**	**Slovenia**	**15,429**	**18**	**2015–2017**	**166.9**
Germany	317	18-24	2008–2011	180.2	**Serbia**	**1017**	**20–29**	**2013**	**166.6**
Latvia	342	20–29	2014	180.2	Belarus	344	18–29	2016–2017	166.6
**Slovenia**	**15,112**	**18**	**2015–2017**	**180.2**	Czechia (Brno)	1213	18–19	2015–2016	166.5
Norway (conscripts)	18,297	~18–19	2011	180.0	**Croatia**	**360**	**18**	**2006–2008**	**166.3**
Hungary	4737	18–25	2016	179.9	Belgium	455	18–30	2013	166.2

* The study measured 211 males and 215 females aged 20–21 years, but the mean height of 21-year-olds was extrapolated from the growth curve.

## Data Availability

Not applicable.
