# Correction: Grasgruber et al. Mapping the Mountains of Giants: Anthropometric Data from the Western Balkans Reveal a Nucleus of Extraordinary Physical Stature in Europe. *Biology* 2022, *11*, 786

**DOI:** 10.3390/biology11071050

**Published:** 2022-07-13

**Authors:** Pavel Grasgruber, Bojan Mašanović, Stipan Prce, Stevo Popović, Fitim Arifi, Duško Bjelica, Dominik Bokůvka, Jan Cacek, Ivan Davidović, Jovan Gardašević, Eduard Hrazdíra, Sylva Hřebíčková, Pavlína Ingrová, Predrag Potpara, Nikola Stračárová, Gregor Starc, Nataša Mihailović

**Affiliations:** 1 Faculty of Sports Studies, Masaryk University, 625 00 Brno, Czech Republic; bokuvka@fsps.muni.cz (D.B.); jan.cacek@gmail.com (J.C.); hrazdira@fsps.muni.cz (E.H.); hrebickova@fsps.muni.cz (S.H.); nikola@2d.cz (N.S.); 2 Faculty for Sport and Physical Education, University of Montenegro, 81400 Niksić, Montenegro; stevo.popovic@ucg.ac.me (S.P.); dbjelica@ucg.ac.me (D.B.); jovan@ucg.ac.me (J.G.); pid.p9193@gmail.com (P.P.); 3 Western Balkan Sport Innovation Lab, 81000 Podgorica, Montenegro; 4 Gimnazija Metković, 20350 Metković, Croatia; stipan.prce@gimnazija-metkovic.net; 5 Faculty for Sport and Physical Education, University of Tetovo, 1200 Tetovo, North Macedonia; fitim_arifi@yahoo.com; 6 Kosovo Olympic Academy, 10 000 Prishtina, Kosovo; 7 Srednja ekonomsko-ugostiteljska škola (Secondary School of Economics and Catering), 85000 Bar, Montenegro; davidovicivan92@yahoo.com; 8 Department of Anthropology, Faculty of Science, Masaryk University, 602 00 Brno, Czech Republic; ingrova.p@mail.muni.cz; 9 Faculty of Sport, University of Ljubljana, 1000 Ljubljana, Slovenia; gregor.starc@fsp.uni-lj.si; 10 Department of Biostatistics, Institute of Public Health, 34000 Kragujevac, Serbia; natalimihailovic@gmail.com

Note: The authors apologize for the rather extensive text corrections. All the shortcomings listed below arose during the final editing of the article and due to the serious health indisposition of the first author (Pavel Grasgruber), it was not possible to resolve them in time.

## 1. Authors’ Names

In the original publication [1], the authors’ surnames were listed without diacritics. At the same time, Natasa M. Mihailovic should not have a middle name. The correct names (with corrected affiliations) appear below.

Pavel Grasgruber ^1,^*, Bojan Mašanović ^2,3,^*, Stipan Prce ^4^, Stevo Popović ^2,3^, Fitim Arifi ^5,6^, Duško Bjelica ^2^, Dominik Bokůvka ^1^, Jan Cacek ^1^, Ivan Davidović ^7^, Jovan Gardašević ^2^, Eduard Hrazdíra ^1^, Sylva Hřebíčková ^1^, Pavlína Ingrová ^8^, Predrag Potpara ^2^, Nikola Stračárová ^1^, Gregor Starc ^9^ and Nataša Mihailović ^10^

## 2. Additional Affiliation

In the published publication, there was a missing affiliation for Bojan Mašanović and Stevo Popović: Western Balkan Sport Innovation Lab, 81000 Podgorica, Montenegro. The affiliations did not include diacritics. There was a typing error in “Ekonomska škola Bar”. The name of the school was not complete.

The corrected affiliations are listed below:

^1^  Faculty of Sports Studies, Masaryk University, 625 00 Brno, Czech Republic; bokuvka@fsps.muni.cz (D.B.); jan.cacek@gmail.com (J.C.); hrazdira@fsps.muni.cz (E.H.); hrebickova@fsps.muni.cz (S.H.); nikola@2d.cz (N.S.)
^2^  Faculty for Sport and Physical Education, University of Montenegro, 81400 Niksić, Montenegro; stevo.popovic@ucg.ac.me (S.P.); dbjelica@ucg.ac.me (D.B.); jovan@ucg.ac.me (J.G.); pid.p9193@gmail.com (P.P.)
^3^  Western Balkan Sport Innovation Lab, 81000 Podgorica, Montenegro
^4^  Gimnazija Metković, 20350 Metković, Croatia; stipan.prce@gimnazija-metkovic.net
^5^  Faculty for Sport and Physical Education, University of Tetovo, 1200 Tetovo, North Macedonia; fitim_arifi@yahoo.com
^6^  Kosovo Olympic Academy, 10 000 Prishtina, Kosovo
^7^  Srednja ekonomsko-ugostiteljska škola (Secondary School of Economics and Catering), 85000 Bar, Montenegro; davidovicivan92@yahoo.com
^8^  Department of Anthropology, Faculty of Science, Masaryk University, 602 00 Brno, Czech Republic; ingrova.p@mail.muni.cz
^9^  Faculty of Sport, University of Ljubljana, 1000 Ljubljana, Slovenia; gregor.starc@fsp.uni-lj.si
^10^  Department of Biostatistics, Institute of Public Health, 34000 Kragujevac, Serbia; natalimihailovic@gmail.com

## 3. Figure/Table Legend

(1) In the original publication, there was a mistake in the legend for Figure 2:

For Zagreb, see Petranović et al. [15].

The correct legend appears below:

For Zagreb, see Petranović et al. [16].

(2) In the original publication, there was a mistake in the legend for Figure 3:

Economic development in the Western Balkans (gross domestic product per capita, by purchasing power parity), compared with the Netherlands and the USA. Source: [23].

The correct legend appears below:

Economic development in the Western Balkans (gross domestic product per capita, by purchasing power parity), compared with the Netherlands and the USA. Source: [24].

(3) In the original publication, there was a mistake in the legend for Figure 4:

The quality of nutrition in the Western Balkans (expressed as the ‘protein index’), compared with the Netherlands and the USA. Source: [24].

The correct legend appears below:

The quality of nutrition in the Western Balkans (expressed as the ‘protein index’), compared with the Netherlands and the USA. Source: [25].

(4) In the original publication, there was a mistake in the legend for Figure 6:

Regional differences in male height within Albania, according to different age categories: 17–25 years (*n* = 1273), 20–39 years (*n* = 2502), and 40–59 years (*n* = 2072). Source: [8].

The correct legend appears below:

Regional differences in male height within Albania, according to different age categories: 17–25 years (*n* = 1273), 20–39 years (*n* = 2502), and 40–59 years (*n* = 2072). Source: [9].

(5) In the original publication, there was a mistake in the legend for Table 1:

“D, standard deviation”.

The correct legend appears below:

“SD, standard deviation”.

## 4. Error in Table

In the original publication, there was a mistake in Table 1. The labels in the table were incorrectly formatted (the names of regions were not aligned to the right). A standard error was added to the row “Montenegro—Central region”. 

The corrected Table 1 appears below.

**Table 1:** Mean height in the former Yugoslavia and Albania (in similar age categories).

Country/Region	Year	Age	Males				Females			
			*n*	Mean Height (cm)	SD	SE	*n*	Mean Height (cm)	SD	SE
**Montenegro**	**2013**	**17–20**	**981**	183.4 (182.9 *)	**6.9**	**0.22**	**1107**	169.4 (168.8 *)	**6.4**	**0.19**
*Central region*			664	183.6	7.0	0.27	711	169.7	6.3	0.24
**Coastal Croatia**	**2015–2017**	**17–20**	**1803**	182.7 (182.6 *)	**6.7**	**0.16**	**782**	167.4 (168.0 *)	**6.2**	**0.22**
*Dalmatia*			1143	183.6 (183.7 *)	6.7	0.20	279	168.8 (168.5 *)	6.0	0.36
**Bosnia & Herzegovina**	**2015–2016**	**17–20**	**3192**	181.7 (181.2 *)	**6.8**	**0.12**	**69**	**169.4**	**6.0**	**0.72**
*Bosnia*			2209	180.9 (180.8 *)	6.5	0.14				
*Herzegovina*			983	183.6 (183.4 *)	6.9	0.22				
**Serbia**	**2013**	**17–25**	**724**	180.7 (180.7 *)	**7.4**	**0.27**	**787**	166.8 (166.8 *)	**6.4**	**0.23**
*Beograd*			155	181.8	7.3	0.59	184	168.3	6.5	0.48
*Šumadija* & *W. Serbia*			225	181.1	7.6	0.51	225	167.4	6.2	0.41
*Vojvodina*			154	180.2	7.4	0.60	194	166.1	6.2	0.45
*South* & *East Serbia*			190	179.8	7.3	0.53	184	165.2	6.5	0.48
**Slovenia**	**2015–2017**	**18**	**15,112**	**180.2**	**6.8**	**0.06**	**15,429**	**166.9**	**6.1**	**0.05**
**Croatia: Zagreb**	**2010**	**18–19**	**131**	180.1 **			**111**	165.2 **		
**Kosovo**	**2016**	**18–20**	**830**	**179.5**	**6.0**	**0.21**	**793**	**165.7**	**4.9**	**0.17**
**North Macedonia**	**2012**	**18**	**596**	**177.4**	**6.5**	**0.27**	**552**	**164.5**	**6.2**	**0.26**
**Albania**	**2017–2018**	**17–25**	**1273**	174.3 (174.4 *)	**6.7**	**0.19**	**2886**	161.5 (161.6 *)	**6.5**	**0.12**

SD: standard deviation; SE: standard error. * A weighted mean considering the population size of individual regions. ** A weighted mean of 18 and 19-year-olds in Zagreb.

## 5. Text Correction

(1) There was an error in the Abstract of the original publication.

This anthropological synthesis includes the measurements of 47,158 individuals (24,642 males and 22,516 females) from the period 2010–2018 and maps detail regional differences in male stature in the Western Balkans.

A correction has been made:

This anthropological synthesis includes the measurements of 47,158 individuals (24,642 males and 22,516 females) from the period 2010–2018 and describes detailed regional differences in male stature in the Western Balkans.

(2) There was an error in the Abstract of the original publication.

... and 18-year-old boys from Dalmatia are even taller (183.7 cm).at a regional level.

A correction has been made:

... and 18-year-old boys from Dalmatia are even taller (183.7 cm) at a regional level.

(3) The text has been modified in the sentence:

This belt also includes two notable regional anomalies with mean heights above 185 cm—one is centered around Široki Brijeg in Western Herzegovina and Central Dalmatia ...

A correction has been made:

This belt also includes two notable regional anomalies with mean heights above 185 cm. One is centered around Široki Brijeg in Western Herzegovina and Central Dalmatia ...

(4) The text has been modified in the sentence:

This increase in stature is driven by several key factors that are closely associated with the rising GDP (gross domestic product) per capita, better nutrition (mainly high-quality proteins from milk, pork, and eggs), ...

A correction has been made:

This increase in stature is driven by several key factors that are closely associated with the rising GDP (gross domestic product) per capita: better nutrition (mainly high-quality proteins from milk, pork, and eggs), ...

(5) There was an error in the reference in the sentence:

In fact, a regression model of six socio-economic and three nutritional variables in 119 countries [17] ...

A correction has been made:

In fact, a regression model of six socio-economic and three nutritional variables in 119 countries [18] ...

(6) The text has been modified in the sentence:

These numbers remain practically the same even after the addition of the Gini index ...

A correction has been made:

These numbers remain practically the same even in a smaller sample of 96 countries, after the addition of the Gini index ...

(7) There were errors in the references in the paragraph:

These eccentric results are easy to understand, when we consider that the GDP in the Netherlands for 2013 was 49,242 USD per capita, whereas that of Montenegro was more than three-times lower (14,870 USD per capita), and the GDP in Bosnia and Herzegovina reached only 12,011 USD per capita in 2015 [23] (Figure 3). Even more important are the statistics of dietary protein quality, assessed using the FAOSTAT database [24]. Indeed, the ‘protein index’ (a ratio between the daily supply of proteins from dairy and pork/wheat), which is the strongest nutritional predictor of height in 44 European countries (r = 0.62, *p* < 0.001; see [17]), is mostly very low in the Western Balkans (Figure 4).

A correction has been made:

These eccentric results are easy to understand, when we consider that the GDP in the Netherlands for 2013 was 49,242 USD per capita, whereas that of Montenegro was more than three-times lower (14,870 USD per capita), and the GDP in Bosnia and Herzegovina reached only 12,011 USD per capita in 2015 [24] (Figure 3). Even more important are the statistics of dietary protein quality, assessed using the FAOSTAT database [25]. Indeed, the ‘protein index’ (a ratio between the daily supply of proteins from dairy and pork/wheat), which is the strongest nutritional predictor of height in 44 European countries (r = 0.62, *p* < 0.001; see [18]), is mostly very low in the Western Balkans (Figure 4).

(8) The text has been modified in the sentence:

... and this is especially true for J-M304 (r = − 0.88, *p* = 0.008), which is also the strongest correlate of shortness in Europe and the Near East (r = −0.86, *p* < 0.001). In accordance with these findings, the inclusion of I-M170 and the Near Eastern Y haplogroups ...

A correction has been made:

This is especially true for J-M304 (r = −0.88, *p* = 0.008), which is also the strongest correlate of shortness in Europe and the Near East (r = −0.86, *p* < 0.001). In accordance with these findings, the inclusion of I-M170 and the three Near Eastern Y haplogroups ...

(9) There were errors in the references in the sentences:

... from adjusted R^2^ = 0.426 to 0.721 [17]. Other European Y haplogroups have a more restricted geographical distribution but they also show geographical relationships with height (see Figure 5A,B and [19]).

A correction has been made:

... from adjusted R^2^ = 0.426 to 0.721 [18]. Other European Y haplogroups have a more restricted geographical distribution but they also show geographical relationships with height (see Figure 5A,B and [20]).

(10) There were errors in the references in the paragraph:

Y haplogroup I-M170 generally constitutes a very interesting case because its origin is very old and can be traced as far back as to the Upper Paleolithic Gravettian culture [25]. I-M170 was also closely associated with the post-glacial expansion of Epigravettian (Late Gravettian) groups from the refugium around the Adriatic sea and became the predominant Y haplogroup in Mesolithic Europe [26]. At present, it reaches a global frequency peak in Herzegovina (70.9%) [27], where it is overwhelmingly represented by the Balkan sub-branch I2a1a-P37.2 and particularly by downstream mutations of I2a1a2-M423 [28]. This dominance of I2a1a2-M423 would result from a relatively recent (possibly even post-Neolithic) founder effect [27–30].

A correction has been made:

Y haplogroup I-M170 generally constitutes a very interesting case because its origin is very old and can be traced as far back as to the Upper Paleolithic Gravettian culture [26]. I-M170 was also closely associated with the post-glacial expansion of Epigravettian (Late Gravettian) groups from the refugium around the Adriatic sea and became the predominant Y haplogroup in Mesolithic Europe [27]. At present, it reaches a global frequency peak in Herzegovina (70.9%) [28], where it is overwhelmingly represented by the Balkan sub-branch I2a1a-P37.2 and particularly by downstream mutations of I2a1a2-M423 [29]. This dominance of I2a1a2-M423 would result from a relatively recent (possibly even post-Neolithic) founder effect [28–31].

(11) There were errors in the references in the paragraph:

Still, the history of I-M170 in the Dinaric Alps remains enigmatic and due to the lack of well-preserved skeletal material, it will be difficult to capture its evolution over time. In fact, no prehistoric samples from Bosnia and Herzegovina and Montenegro have been analyzed so far [31], and to our knowledge, the oldest occurrence of I-M170 (I2a1a2-M423) in the Dinaric area was documented in the Bezdanjaˇca Cave (Lika-Senj county, Adriatic Croatia) and was dated to ~1200 cal. BC [32]. Quite recently, Utevska [33] even doubted the local origin of I2a1a-P37.2 in the Western Balkans and hypothesized that it had expanded ~3000 years ago from the area east of the Carpathians.

A correction has been made and the text has been modified:

Still, the history of I-M170 in the Dinaric Alps remains enigmatic and due to the scarcity of well-preserved skeletal material, it will be difficult to capture its evolution over time. In fact, the website of Ancient Human DNA [32] does not register a single prehistoric sample from Bosnia and Herzegovina and Montenegro [32]. To our knowledge, the oldest occurrence of I-M170 (I2a1a2-M423) in the Dinaric area was documented in the Bezdanjača Cave (Lika-Senj County, Adriatic Croatia) and was indirectly dated to ~1200 cal. BC [33]. A more recent analysis of Bronze Age individuals from this cave reportedly found another case of I2a1a2-M423 (I. Lazaridis, personal communication). However, Utevska [34] doubted the local origin of I2a1a-P37.2 in the Western Balkans and hypothesized that it had expanded ~3000 years ago from the area east of the Carpathians.

(12) There was an error in the reference in the sentence:

Environmental explanations fail especially in Montenegro, where people in the richer coastal regions around the capital of Podgorica are much shorter than those in the remote mountain areas of Kolašin and Šavnik lying mostly on flysch sediments [34].

A correction has been made:

Environmental explanations fail especially in Montenegro, where people in the richer coastal regions around the capital of Podgorica are much shorter than those in the remote mountain areas of Kolašin and Šavnik lying mostly on flysch sediments [35].

(13) There was an error in the reference in the sentence:

In contrast, Elbasan and Kukës remained the poorest counties in Albania [35].

A correction has been made:

In contrast, Elbasan and Kukës remained the poorest counties in Albania [36].

(14) There was an error in the reference in the sentence:

Comparisons of this sort are already available thanks to genome-wide association studies (GWAS) and have shown that height is highly polygenic—depending on the combination of a large number of genes, each of which explains only a very small part of the total variability [36,37].

A correction has been made:

Comparisons of this sort are already available thanks to genome-wide association studies (GWAS) and have shown that height is highly polygenic—depending on the combination of a large number of genes, each of which explains only a very small part of the total variability [37,38].

(15) There were errors in the references in the sentences:

However, the identification of such SNPs is difficult due to the confounding role of environment [38]. Although the accuracy of the polygenic height scores has been improving at the individual level [39], their success at the population level is still rather mixed because height-associated SNPs are population-specific and most GWAS have been performed on Europeans [40,41].

A correction has been made:

However, the identification of such SNPs is difficult due to the confounding role of environment [39]. Although the accuracy of the polygenic height scores has been improving at the individual level [40], their success at the population level is still rather mixed because height-associated SNPs are population-specific and most GWAS have been performed on Europeans [41,42].

(16) There was an error in the reference in the sentence:

As a result, the number of publicly available individual genomes from the Western Balkans is relatively small and considering that they are not sorted according to regions in the POPRES database [42], a direct testing of the genetic hypothesis was not possible.

A correction has been made:

As a result, the number of publicly available individual genomes from the Western Balkans is relatively small and considering that they are not sorted according to regions in the POPRES database [43], a direct testing of the genetic hypothesis was not possible.

(17) There were errors in the references in the paragraph:

Contemporary genetic studies aimed at the evolution of height in Europe (e.g., [38,40,43]) touched the Western Balkan region only superficially and are not very helpful either. Their results generally predict tall statures in ancient populations emerging out of the Epigravettian refugium (genetic cluster Villabruna/WHG) and in the nomadic Eneolithic cultures from the East European steppe, and attribute low genetic predispositions to Near Eastern agriculturalists. This result agrees with the Y chromosomal picture but curiously, polygenic height scores in the modern Western Balkan populations are only moderate or below average. For example, the preprint by Berg et al. [40] estimated moderate values in present-day Croats and predicted the highest values in Icelanders, Englishmen, and Scots. English and Scottish males reach only ~178 cm, despite a long historical lead in industrial development, whereas Croats are almost 3 cm taller.

A correction has been made and the text has been modified:

Contemporary genetic studies aimed at the evolution of height in Europe (e.g., [39,41,44]) touched the Western Balkan region at best only superficially and are not very helpful either. Their results generally predict tall statures in ancient populations emerging out of the Epigravettian refugium (genetic cluster Villabruna/WHG) and in the nomadic Eneolithic cultures from the East European steppe (genetic cluster Yamnaya), and attribute low genetic predispositions to Near Eastern agriculturalists. This result agrees with the Y chromosomal picture but curiously, polygenic height scores in the modern Western Balkan populations are only moderate or below average. For example, the preprint by Berg et al. [41] estimated medium height in present-day Croats and predicted the highest values in Icelanders, Englishmen, and Scots. At the same time, English and Scottish males reach only ~178 cm, despite a long historical lead in industrial development, whereas Croats are almost 3 cm taller.

(18) There was an error in the reference in the sentence:

Sohail et al. [38] used presumably unconfounded SNPs based on British individuals from the UK Biobank, but these markers also produced only low-to-medium polygenic scores in a small POPRES sample from the former Yugoslavia (*n* = 44).

A correction has been made:

Sohail et al. [39] used presumably unconfounded SNPs based on British individuals from the UK Biobank, but these markers also produced only low-to-medium polygenic scores in a small POPRES sample from the former Yugoslavia (*n* = 44).

(19) There were errors in the paragraph:

In fact, the projection of correlation lines between male height and the frequencies of I-M170 indicates that well-nourished males in Herzegovina and southern Dalmatia could potentially reach an astonishing average height of ~190 cm [11,12]. This value, however seemingly improbable, is already not too far from the urban means that we documented in Makarska (187.6 cm), Imotski (186.2 cm), and Čapjina (185.9 cm). The current development of dietary protein quality appears the most optimistic in the case of Croatia and Montenegro (Figure 4). Judging from the measurements of recruits in the capital of Podgorica born during the 1960s [44], the height of Montenegrin men has been increasing at a rate of 1.7 cm/decade. Croatian boys measured during nationwide surveys in 1980–1984 and 2006–2008 grew by 2.9 cm [45], until their growth stopped (or even reversed) during the economic recession in the late 1990s [46]. The current positive trend in the dietary protein quality predicts that their height should increase again.

A correction has been made:

In fact, the projection of correlation lines between male height and the frequencies of I-M170 indicates that well-nourished males in Herzegovina and southern Dalmatia could potentially reach an astonishing average height of ~190 cm [12,13]. This value, however seemingly improbable, is already not too far from the urban means that we documented in Makarska (187.6 cm), Imotski (186.2 cm), and Čapljina (185.9 cm). The current development of dietary protein quality appears the most optimistic in the case of Croatia and Montenegro (Figure 4). Judging from the measurements of recruits in the capital of Podgorica born during the 1960s [45], the height of Montenegrin men has been increasing at a rate of 1.7 cm/decade. Croatian boys measured during nationwide surveys in 1980–1984 and 2006–2008 grew by 2.9 cm [46], until their growth stopped (or even reversed) during the economic recession in the late 1990s [47]. The current positive trend in the dietary protein quality predicts that their height should increase again.

(20) There was an error in the reference in the sentence:

Provided that all the lagging Albanian regions reach sufficiently high nutritional and socio-economic standards, we expect that the north-to-south gradient in height, which was once reported by Coon [4], will emerge again, and the height difference between Albania and Montenegro should also decrease.

A correction has been made:

Provided that all the lagging Albanian regions reach sufficiently high nutritional and socio-economic standards, we expect that the north-to-south gradient in height, which was once reported by Coon [6], will emerge again, and the height difference between Albania and Montenegro should also decrease.

(21) There was an error in the reference in the sentence:

Given that Albanians already consume the highest amount of dairy proteins in the world [24], the values of the ‘protein index’ in Albania are unlikely to rise much higher.

A correction has been made:

Given that Albanians already consume the highest amount of dairy proteins in the world [25], the values of the ‘protein index’ in Albania are unlikely to rise much higher.

(22) There was an error in the reference in the sentence:

Nevertheless, 18-year-old boys from the high schools in Tuzla (Bosnia and Herzegovina) grew by 2 cm between 1980 and 2003, despite war hardships [47], and judging from our data, their height has further increased from 178.8 cm in 2003 to 180.5 cm in 2015.

A correction has been made:

Nevertheless, 18-year-old boys from the high schools in Tuzla (Bosnia and Herzegovina) grew by 2 cm between 1980 and 2003, despite war hardships [48], and judging from our data, their height has further increased from 178.8 cm in 2003 to 180.5 cm in 2015.

## 6. Author Contributions

The names in Author Contributions did not include diacritics.

**Author Contributions:** P.G. and B.M. co-wrote the manuscript. S.P. (Stipan Prce), S.P. (Stevo Popović) and G.S. approved the final version. F.A., D.B. (Duško Bjelica), D.B. (Dominik Bokůvka), J.C., I.D., J.G., E.H., S.H., P.I., P.P., N.S. and N.M. directly collaborated on the field research, or worked as managers. All authors have read and agreed to the published version of the manuscript.

The authors apologize for any inconvenience caused and state that the scientific conclusions are unaffected. This correction was approved by the Academic Editor. The original publication has also been updated.
